# Reduced partitioning of plastic strain for strong and yet ductile precipitate-strengthened alloys

**DOI:** 10.1038/s41598-018-26917-0

**Published:** 2018-06-06

**Authors:** R. D. Jones, F. Di Gioacchino, H. Lim, T. E. J. Edwards, C. Schwalbe, C. C. Battaile, W. J. Clegg

**Affiliations:** 1 Gordon Laboratory, Department of Materials Science and Metallurgy, The University of Cambridge, 27 Charles Babbage Rd, Cambridge, CB3 0FS UK; 2 Department of Computational Materials and Data Science, Sandia National Laboratories, Albuquerque, NM 87185 United States

## Abstract

When a material that contains precipitates is deformed, the precipitates and the matrix may strain plastically by different amounts causing stresses to build up at the precipitate-matrix interfaces. If premature failure is to be avoided, it is therefore essential to reduce the difference in the *plastic* strain between the two phases. Here, we conduct nanoscale digital image correlation to measure a new variable that quantifies this plastic strain difference and show how its value can be used to estimate the associated interfacial stresses, which are found to be approximately three times greater in an Fe-Ni_2_AlTi steel than in the more ductile Ni-based superalloy CMSX-4^®^. It is then demonstrated that decreasing these stresses significantly improves the ability of the Fe-Ni_2_AlTi microstructure to deform under tensile loads without loss in strength.

## Introduction

Many alloys used in the most demanding structural applications are made up of nanosized precipitates dispersed within a metallic matrix^1–11^. The precipitates strengthen the solid by resisting the movement of the defects that give rise to plastic deformation. However, if there is a difference in *plastic* strain between the matrix and the precipitates, stresses are generated at the precipitate-matrix interfaces that can lead to premature failure^12–14^. While X-ray and neutron diffraction techniques have been used to quantify the elastic strain difference between the two phases^15–20^, it has not so far been possible to measure this plastic strain difference in these materials. The present paper demonstrates a way of doing this and describes how to estimate the interfacial stresses from such measurements. Also, it shows that reducing these stresses improves the usually poor ductility of very strong precipitate-matrix microstructures. This is significant, as the latter must be able to deform plastically under tensile loads to prevent the nucleation of damage and the propagation of cracks^21^.

The strain that accumulates at the surface of a testpiece can be quantified using the expression for the maximum shear strain, *γ*_*max*_, which is a positive scalar-valued function of the strain components, as shown in the Methods section. As the elastic contribution to the total strain is negligible at large deformation, the partitioning of plastic strain can be simply expressed as the difference between the mean values of *γ*_*max*_, in the matrix, $\langle \,{\gamma }_{max}^{M}\rangle$, and the precipitates, $\langle {\gamma }_{max}^{P}\rangle$:

1 $${p}_{plastic}=\langle {\gamma }_{max}^{M}\rangle -\langle {\gamma }_{max}^{P}\rangle $$

The value of *p*_*plastic*_ is positive if the strain is less in the precipitates than in the matrix and negative if it is greater in the precipitates.

To measure $\langle {\gamma }_{max}^{M}\rangle$ and $\langle {\gamma }_{max}^{P}\rangle$ in Equation 1, one must be able to map strains at the length scale of the precipitates. This can be achieved using digital image correlation (DIC) to track the displacement of fine speckles applied onto the area of interest before testing^22^. The speckles must be present in both the precipitates and the matrix channels, which can be even narrower than the precipitate size when the volume fraction occupied by the matrix is greater than about one-eighth (having considered cubic shaped precipitates). After mapping the strains, masking of the precipitates is required to group the strain data by phase type. Therefore, the precipitates must be visible in the same images used for DIC. In precipitate-strengthened alloys with nanosized precipitates, the speckles must then be of nanoscale size and applied with a method that distributes them evenly (i.e. not randomly) across the area to maximise both the spatial resolution and the accuracy of the DIC. This contrasts with the less stringent requirements to be met when characterising the distribution of plastic strain in polycrystalline multiphase alloys that show comparatively large and distant second phase grains or particles^23–30^.

Example of high resolution DIC measurements of plastic strain in the Ni-based precipitate-strengthened alloy René 88DT can be found in the work of Stinville *et al*.^31,32^. In this, the speckles pattern is created by carving the precipitates exposed at the surface of the sample using etchants. As each carved precipitate represents a speckle, the relative displacements of the speckles obtained by DIC can only be used to quantify the deformation of the matrix material. One can then envisage the use of remodelling of deposited films to create a nanoscale and non-random pattern in a non-destructive fashion^33,34^. However, the residues of deposited material between the speckles would not allow for the visualization of the underlining precipitates.

Here, we use the nanoscale DIC method recently developed to accurately map strains in small-scale testing^35–37^ to measure *p*_*plastic*_ at room temperature in two precipitate-strengthened alloys, the single crystal Ni-based superalloy, CMSX-4^®^, and an Fe-Ni_2_AlTi steel. CMSX-4 is widely used for turbine blades in gas turbine engines and automotive turbochargers. It contains a volume fraction of ~0.7 of γ′-Ni_3_Al, which are ordered precipitates embedded in a Ni-based γ-matrix^1^ of same crystallographic orientation (coherent precipitates). The Fe-Ni_2_AlTi steel shows an analogous microstructure with a ~0.5 volume fraction of coherent Ni_2_AlTi β′-precipitates in an Fe-based β-matrix. These steels have been proposed for applications in supercritical steam turbines and yet their poor ductility has been preventing their practical use^4^.

## Measurements of Plastic Strain Partitioning

To measure *p*_*plastic*_ in CMSX-4 and at the same time demonstrate its independence of the loading direction, two CMSX-4 micropillars were compressed along different crystallographic directions (〈001〉 and 〈011〉). A third micropillar was compressed to measure *p*_*plastic*_ in the Fe-Ni_2_AlTi steel. A fourth micropillar was milled from the ferritic matrix material and tested for comparison.

**Figure 1:**
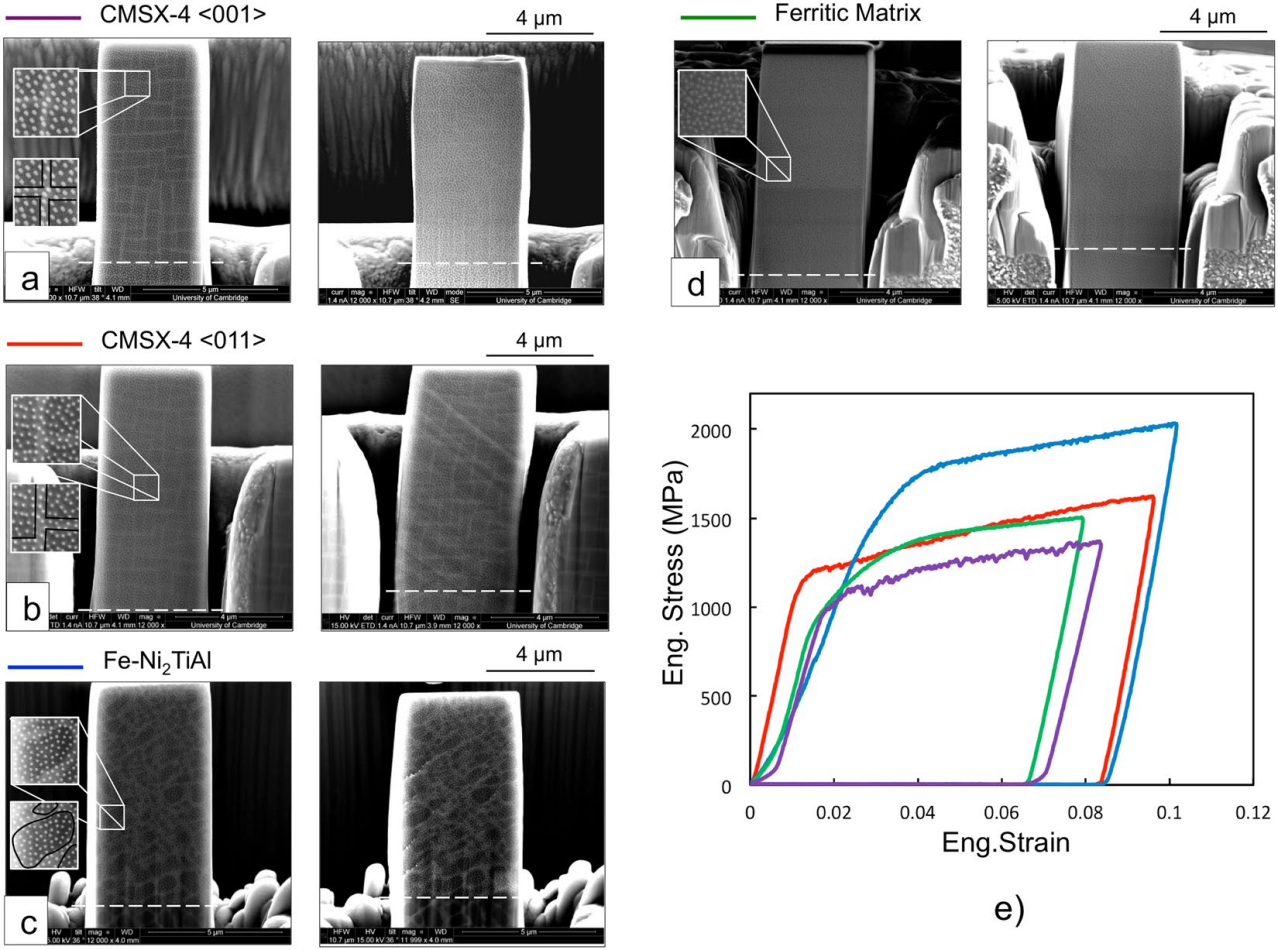
(**a**–**d**) SEM images of the pillars before (left) and after ~0.1 compressive strain (right) with enlarged areas showing the Pt speckle pattern. The white dashed lines, which translate with the base of the pillars, help to visualise the macroscopic strain. The lateral offset of the top part of the CMSX-4 〈011〉 pillar is attributed to the combination of single slip (as seen in the strain map) and the sliding at the pillar-indenter interface. (**e**) Stress-strain curves.

The secondary electron images of the four micropillars with their speckle pattern before and after compression are shown in Fig. 1a–d. As shown in the enlarged areas, the ~40 nm sized and evenly distributed speckles could be imaged in high contrast while also imaging the precipitates. Table 1 includes the estimates of the average size, spacing and volume fraction of the precipitates.

**Table 1:** Microstructural parameters, strain partitioning *p*_*plastic*_ and back stress *τ*_*B*_. The elastic constants are taken from Meyers *et al*.^38^.

Alloy	Vol. Fr.	Size (nm)	Spacing (nm)	Misfit (%)	$\langle {{\boldsymbol{\gamma }}}_{{\boldsymbol{\max }}}^{{\boldsymbol{M}}}\rangle$	$\langle {{\boldsymbol{\gamma }}}_{{\boldsymbol{\max }}}^{{\boldsymbol{P}}}\rangle$	*p*_*plastic*_	*G*_*M*_ (GPa)	*τ*_*B*_ (MPa)
CMSX-4 [010]	0.71	510	160	—	0.1418	0.1450	**−0**.**0032**	123.4	**280**
CMSX-4 [011]	“	“	“	—	0.1442	0.1470	**−0**.**0028**	“	**245**
Fe-Ni_2_AlTi	0.51	340	330	1.5	0.1354	0.1224	**0**.**0130**	116.5	**772**
+1.4 at.% Mo	0.47	150	160	1.1	0.1052	0.1032	**0**.**0020**	“	**108**
+3.3 at.% Mo	0.45	89	110	0.8	0.1282	0.1288	**−0**.**0006**	“	**31**

The stress-strain curves of the micropillar compression tests in Fig. 1e give yield stresses of the order of 1000 MPa and 1100 MPa for the CMSX-4 〈010〉 and CMSX-4 〈011〉, 1500 MPa for the Fe-Ni_2_AlTi and 1000 MPa for the ferritic matrix only. As shown in Methods, these values were multiplied by the Schmid factors to estimate the critical resolved shear stresses. For CMSX-4, a mean value of ~460 MPa is obtained, which is similar to that reported in previous micropillar compression tests^39^. Meanwhile, the Fe-Ni_2_AlTi has a much higher critical stress of ~740 MPa. Without the precipitates, i.e. in the ferritic matrix micropillar, this decreases to ~480 MPa, in line with literature values of quenched low-alloy steels^40^.

The SEM images in the four sets in Fig. 1 were digitally correlated to map the strains across the micropillars. The location of the precipitates was later identified in the maps to extract $\langle {\gamma }_{max}^{M}\rangle$ and $\langle {\gamma }_{max}^{P}\rangle$ in Equation 1, Table 1. The derivation of the experimental error of ~0.0008 associated with the noise in the acquisition of these images is described in the Methods section. To ensure meaningful comparison, measurements were taken over areas that had undergone similar macroscopic strains.

**Figure 2:**
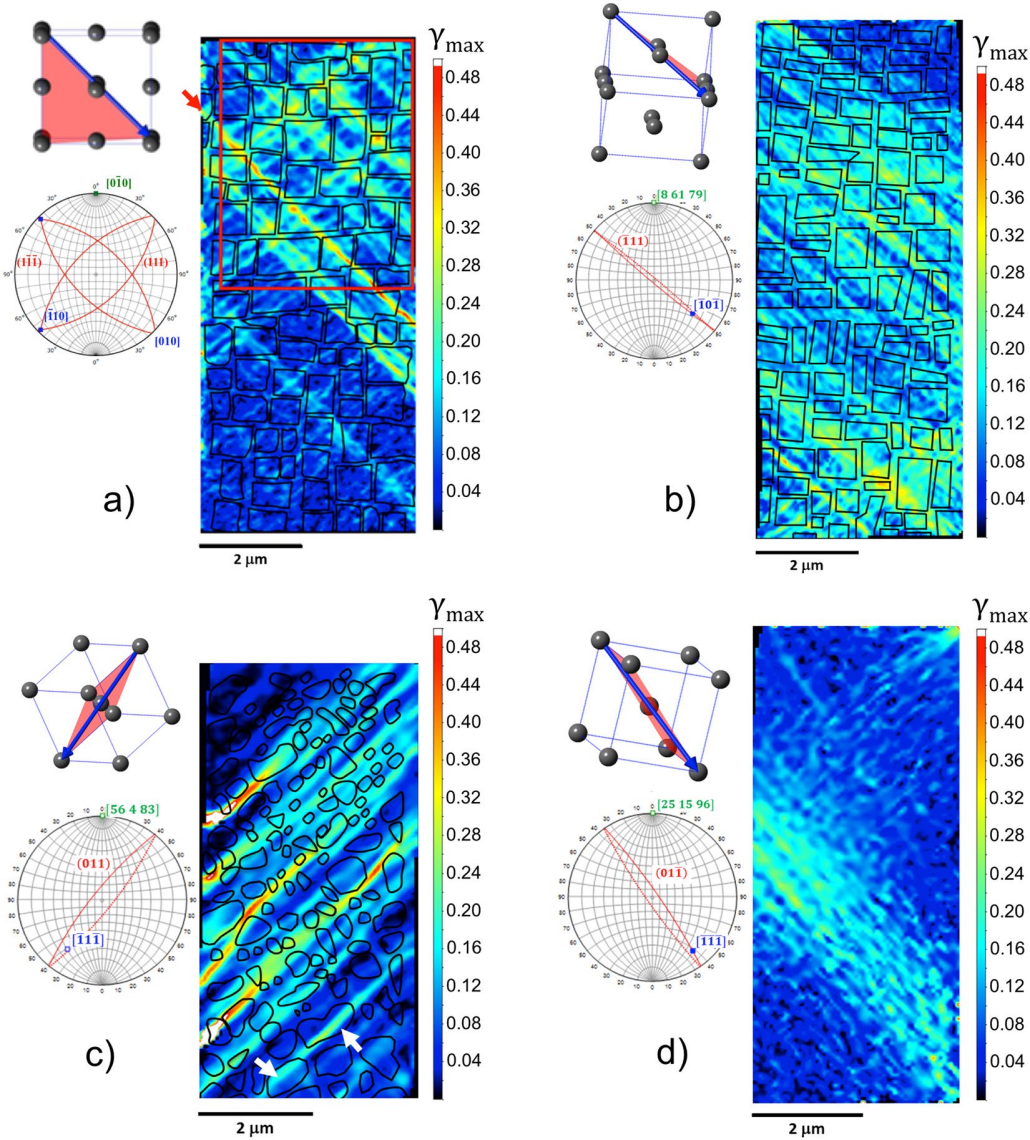
Maximum shear strain maps of the four micropillars with outline of the precipitates. (**a**) 〈001〉 oriented CMSX-4 micropillar. The red line outlines the area for which *p*_*plastic*_ has been measured (**b**) Near 〈011〉 oriented CMSX-4 micropillar. (**c**) Fe-Ni_2_AlTi micropillar. (**d**) Ferritic matrix-only micropillar, i.e. without Ni_2_AlTi precipitates. Unit cells and stereograms show the crystallographic orientation and active slip systems in each pillar. The compression axis is shown in green, the slip planes in red and the slip directions in blue.

The maps of *γ*_*max*_ for the 〈001〉 and the 〈011〉 oriented CMSX-4 micropillars with an outline of the precipitates are shown in Fig. 2a and b, respectively. The strain accumulates along slip bands that align with the traces of the {111}〈011〉 slip systems of highest resolved shear stress and are ~0.25 μm apart, i.e. less than the precipitate size. The shear strain varies along these bands, for instance between ~0.32 and ~0.40 in the most intense band in the strain map of Fig. 2a, with no apparent strain difference between the two phases. However, negative values of *p*_*plastic*_ = −0.0032 and *p*_*plastic*_ = −0.0028 measured for the 〈001〉 and 〈011〉 oriented micropillars, respectively, show that the precipitate phase has strained more than the matrix phase. This result provides evidence in support of early studies^41^ and more recent neutron diffraction measurements^16^ that are consistent with the precipitate phase of CMSX-4 being the soft phase at room temperature. Also, the relatively small difference between the two values, which is within the measurement uncertainty, suggests that the loading direction has no major effect on the partitioning of plastic strain.

The *γ*_*max*_ map of the Fe-Ni_2_AlTi steel in Fig. 2c shows slip bands on the {011}〈111〉 system with the highest resolved shear stress. Many precipitates including the large ones highlighted by white arrows shear with the matrix. However, some large precipitates appear to resist shearing by stopping or deflecting slip bands, as non-deformable particles do^42,43^. This increases the average slip band spacing to ~0.38 μm, a value which is nearly twice the ~0.24 μm measured for the micropillar of the matrix material, Fig. 2d. These precipitates can be easily identified on the map as maximum shear strains of ~0.48 accumulate in the matrix material that surrounds them. This gives a positive *p*_*plastic*_ = 0.0130, the magnitude of which is thus more than four times that measured for CMSX-4.

## Interfacial Back Stresses for the Case of Deformable Particles

Coherency of the precipitates with the matrix in precipitate-strengthened alloys simplifies the treatment of the incompatibility of plastic deformation between the two phases and the derivation of the associated interfacial stresses using analytical models. Here, we propose an extension of the Ashby model^12^ for hard (i.e. non-deformable) particles to the case of (coherent) sheareable particles.

**Figure 3:**
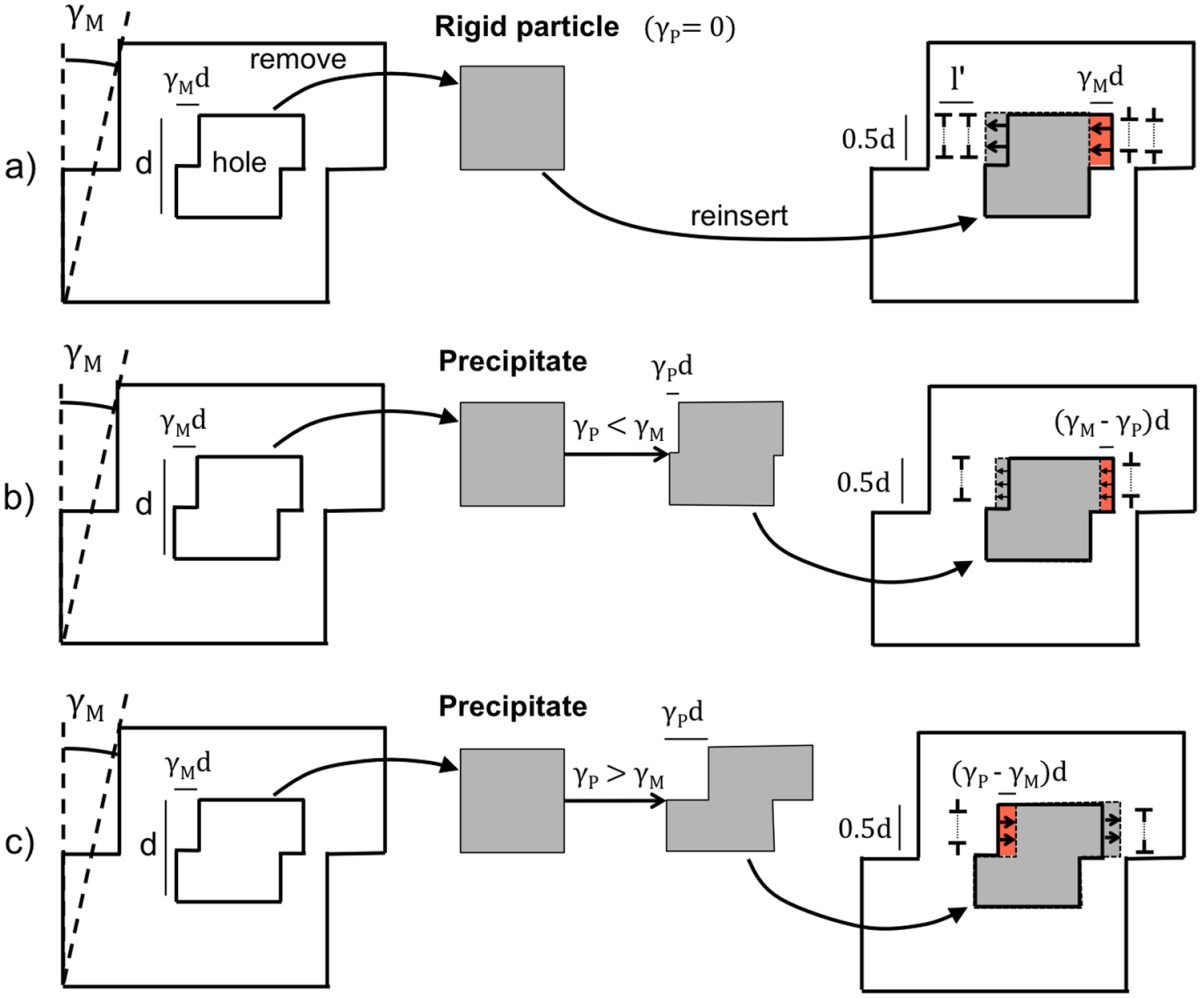
Diagram illustrating the three-steps abstraction of 1-removing the particle, 2- allowing the matrix to deform freely (intermediate configuration) and 3- reinserting the particle to derive a compatible arrangement of GNDs. (**a**) Case for a rigid particle, as in Ashby^12^. (**b**) A precipitate shearing less than the matrix. (**c**) A precipitate shearing more than the matrix.

Taking a unit volume of particle-matrix material, Ashby imagined removing the particle before deformation so that the hole left behind would be free to change shape together with the matrix, Fig. 3a (left). This gives rise to a mismatch in the geometry of the undeformed particle and the hole. Upon reinsertion, a flow of dislocations must then displace the matrix around the particle to prevent any overlap or detachment of material at the particle-matrix interface, Fig. 3a (right). These so-called geometrically necessary dislocations (GNDs)^44^ would pile-up against the precipitate, exerting a back stress that inhibits further slip in the matrix. Although these stresses strengthen the solid, their build-up promotes cavitation at the particle-matrix interfaces under tensile loads, causing internal fracture in particles with large aspect ratios^14^.

There is no unique arrangement of GNDs that restores the hole to its original shape. Figure 3 illustrates the particular case in which this is achieved by inserting *n* prismatic loops of Burgers vector *b* either side of a non-deformable cubic particle of size *d*. Taking *l*′ as the length of each prismatic column and considering isotropic elastic behaviour of the matrix material, Ashby estimated the maximum shear component of the back stress at the surface of the particle, *τ*_*B*_, to be of the order of ${\tau }_{B}=\frac{{G}_{M}nb}{l^{\prime} }$, where *G*_*M*_ is the shear modulus of the matrix (note that a different expression, which includes the square root of the estimated dislocation density, has been given for this stress in Brown and Stobbs^45^). He then linked *l*′ to the precipitate volume fraction $f=\frac{d}{l^{\prime} }$ and obtained:

2 $${\tau }_{B}={G}_{M}f\frac{nb}{d}$$

Geometry shows that the excess volume of matrix material to be inserted or removed by prismatic glide either side of the particle is ${\rm{\Delta }}V=d{\rm{\Delta }}A=0.5\,{V}_{P}{\gamma }_{M}=0.5{d}^{3}{\gamma }_{M}$, where Δ*A* is the area highlighted in red in Fig. 3a and *V*_*P*_ = *d*^3^ is the volume occupied by the particle. Therefore, taking 0.5*d*^2^ as the area bounded by each loop, the excess volume is Δ*V* = 0.5*nbd*^2^. Combining the two expressions for Δ*V* above gives:

3 $$n=\frac{{\gamma }_{M}d}{b}$$

Finally, substituting Equation 3 for *n* in Equation 2 gives:

4 $${\tau }_{B}={G}_{M}f{\gamma }_{M}$$

Equation 4 cannot be used to evaluate *τ*_*B*_ in materials that contain deformable particles because the excess volume Δ*V* would also depend on *γ*_*P*_. However, *γ*_*P*_ can now be experimentally measured. One can then simply imagine having sheared the particle by such known amount before reinserting it, as shown in Fig. 3b and c. If *γ*_*P*_ < *γ*_*M*_, then ${\rm{\Delta }}V=d{\rm{\Delta }}A=0.5{d}^{3}({\gamma }_{M}-{\gamma }_{P})$, Fig. 3b, and ${\tau }_{B}={G}_{M}f({\gamma }_{M}-{\gamma }_{P})$ (assuming the GNDs are still stored in the matrix material). Similarly, if the strain in the particle is greater than that in the matrix, i.e. *γ*_*P*_ > *γ*_*M*_ in Fig. 3c, it is ${\rm{\Delta }}V=d{\rm{\Delta }}A=0.5{d}^{3}({\gamma }_{P}-{\gamma }_{M})$ and ${\tau }_{B}={G}_{M}f({\gamma }_{P}-{\gamma }_{M})$. Ashby’s relation can then be generalised for deformable particles in terms of the strain difference between the two phases as:

5 $${\tau }_{B}={G}_{M}f|{\gamma }_{M}-{\gamma }_{P}|$$

Setting *γ*_*P*_ = 0 recovers the case of a non-deformable particle.

In DIC experiments, the expression for the maximum shear strain is used, as mentioned above and detailed in the Methods section. Moreover, the strain of a given particle or matrix region is obtained as an average of the values measured within their respective domains. Hence, we rewrite Equation 5 as:

6 $${\tau }_{B}=|{G}_{M}f\langle {\gamma }_{max}^{M}\rangle -\langle {\gamma }_{max}^{P}\rangle |={G}_{M}f|{p}_{plastic}|$$

Inserting the values of *G*_*M*_, *f* and *p*_*plastic*_ in Table 1 into Equation 6 gives *τ*_*B*_ = 263 MPa for CMSX-4 and about three times this value, *τ*_*B*_ = 772 MPa, for the Fe-Ni_2_AlTi.

Estimating relatively low back stresses for CMSX-4 is consistent with the 20% elongation that this can achieve at room temperature^46^. On the other hand, the relatively high value obtained for Fe-Ni_2_AlTi material, which may contribute to provide creep resistance at high temperatures as plastic deformation is confined to the matrix, is consistent with its poor ductility. In fact, although exhibiting strengths characteristic of the strongest steels, as shown above, the “as received” Fe-Ni_2_AlTi microstructure does not possess the unique combination of high strength and good ductility that defines the emerging class of so-called ultrastrong (precipitate-strengthened) steels^6,10,11^.

## A Ductile Fe-Ni_2_AlTi Microstructure

**Figure 4:**
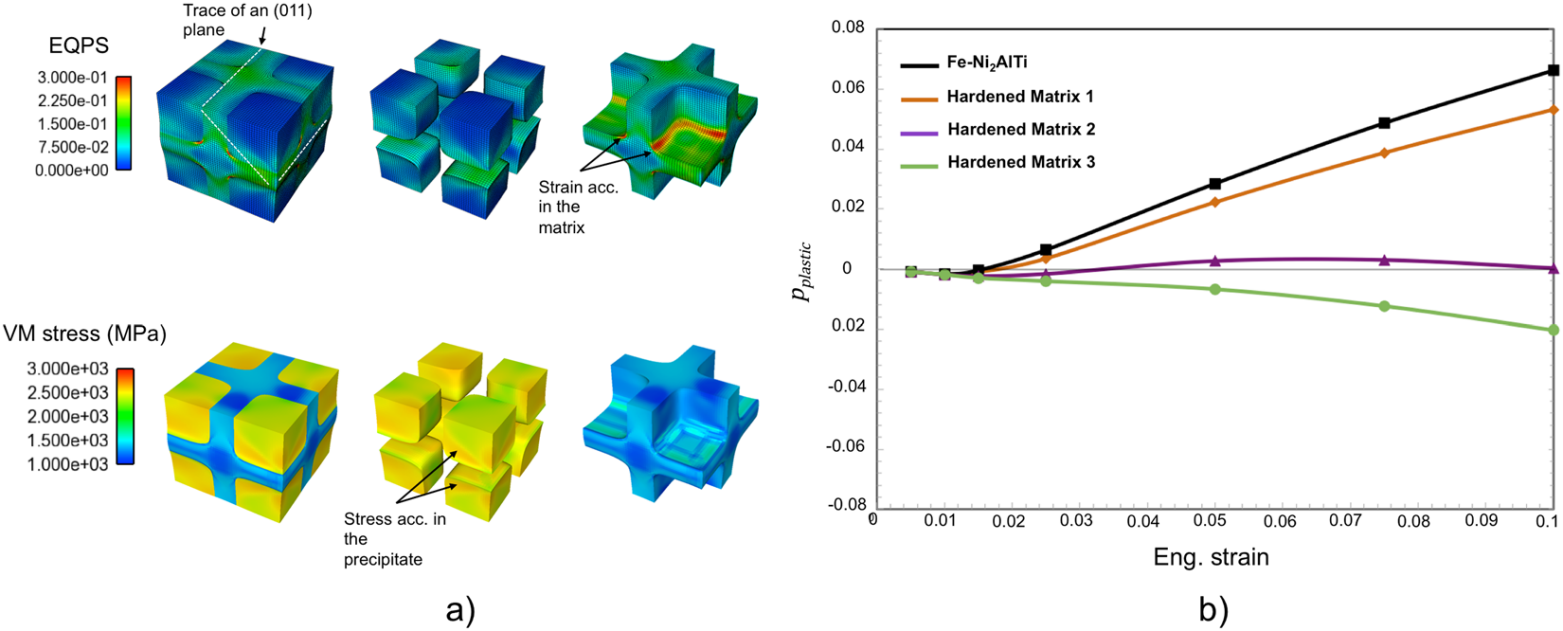
CP-FE simulations. (**a**) Top: von Mises strain (also known as the equivalent plastic strain EQPS) at 0.1 compressive strain shows bands along the {011} planes that cross the edge of the precipitate where strains of ~0.3 accumulate in the matrix. Bottom: von Mises stresses of ~2.5 × 10^+3^ MPa build up at the interface as well and yet on the side of the precipitate. (**b**) Variation of *p*_*plastic*_ in the Fe-Ni_2_AlTi (precipitate-matrix) microstructure.

The finite element method (FEM)^47^ is used to solve the partial differential equations (PDEs) governing the plasticity problem in domains that can be geometrically complex, such as the volume occupied by real particles. Crystal plasticity finite element (CP-FE) calculations, in particular, allow restricting the shear deformation along slip planes and directions and the modelling of the strain hardening associated with the accumulation of dislocations in the crystal lattice^48^. Here, CP-FE analysis is used to gain a preliminary understanding of the factors influencing the evolution of *p*_*plastic*_. To this end, we consider the compression of a precipitate-matrix unit volume of the Fe-Ni_2_AlTi microstructure. Our CP-FE calculations are based on the conventional elastoplastic rate-dependent formulation^49^. The Methods section provides details of the FE model and set-up. Predictions of Von Mises strains and stresses and the variation of *p*_*plastic*_ are shown in Fig. 4a and b, respectively. During the initial elastic loading, *p*_*plastic*_ is negative because the ferritic matrix is stiffer than the Ni_2_AlTi precipitate (values of the elastic constants and hardening parameters are provided in Methods). The matrix then yields and its deformation at the interface with the precipitate forces this to continue straining elastically. Consequently, *p*_*plastic*_ turns positive as plastic strain accumulates in the matrix while stress does so in the precipitate, Fig. 4a. Eventually, the precipitate yields. However, plastic deformation is limited to its edges so that the value of *p*_*plastic*_ keeps rising almost linearly with the applied strain, Fig. 4b.

Increasing the strain hardening rate of the matrix material (as well as softening the Ni_2_AlTi phase) is predicted to bring forward precipitate shearing, leading to a decrease in the extent of plastic strain partitioning. This is shown in the same plot in Fig. 4b by the *p*_*plastic*_ curves (Hardened Matrix 1–3) obtained after having increased the values of the hardening parameters for the ferritic matrix.

A way of softening the precipitates is to reduce the coherency stresses associated with the lattice misfit at the precipitate-matrix interfaces, as this makes it easier for the dislocations in the matrix to reach the interfaces and enter the precipitates^50^. On the other hand, a way of increasing the rate of strain hardening in the matrix is to increase the number density of precipitates, as this proportionally increase the number of obstacles that the dislocations in the matrix need to overcome^51^.

Addition of Mo is known to reduce the lattice misfit and together decrease the size and spacing between the precipitates in the steel^52^. In the present study, the Fe-Ni_2_AlTi steel was alloyed with 1.5 at.% and 5 at.% Mo. Adding Mo led to the formation of an additional Mo-rich hard phase. Lower Mo contents (1.4 at.% and 3.3 at.%, respectively) were therefore found at the length scale of the precipitate-matrix microstructure, where Mo accumulated in the matrix. The reduced values of the precipitates size and spacing and in the lattice misfit are given in Table 1.

**Figure 5:**
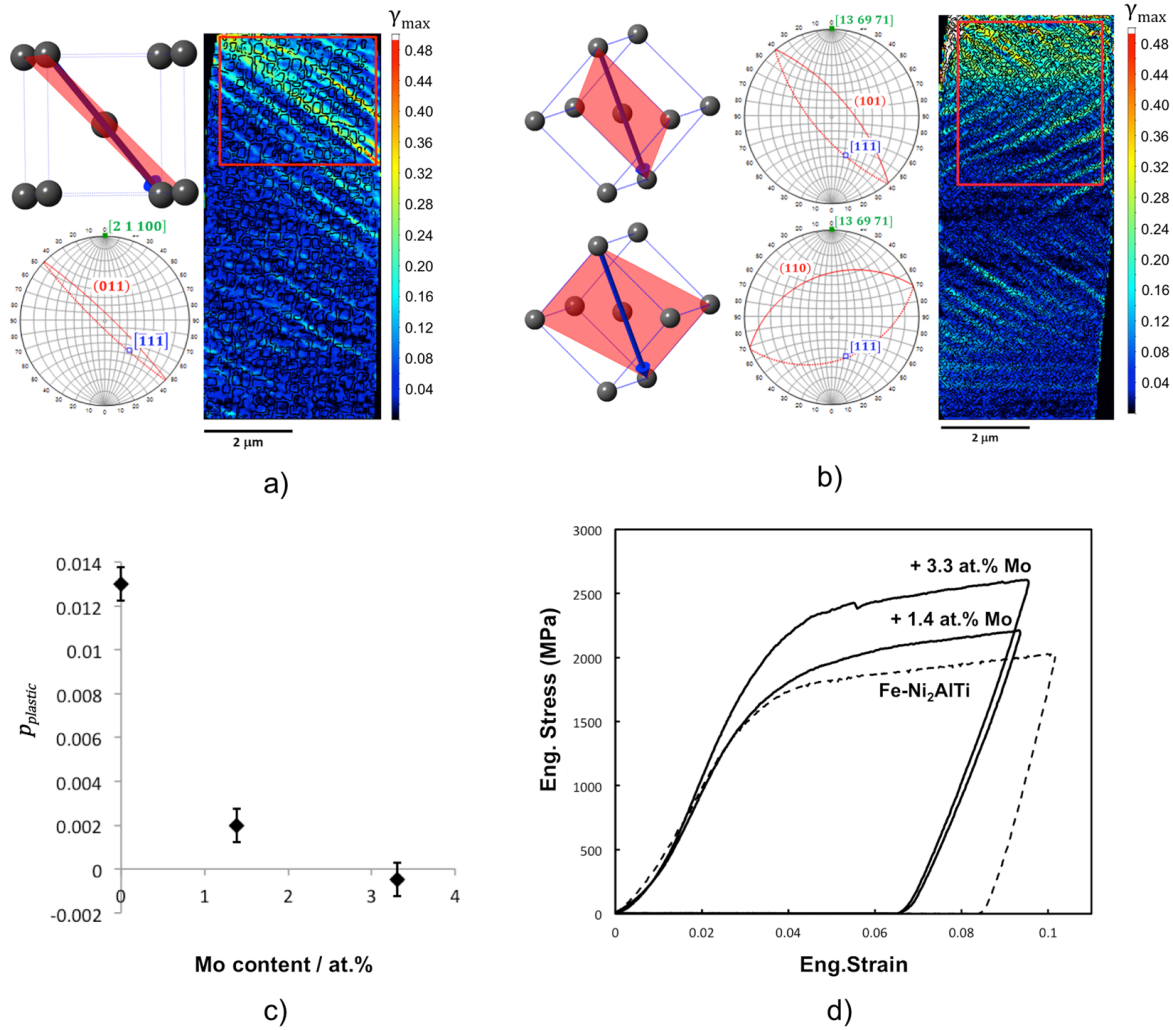
(**a** and **b**) Maximum shear strain maps of the Fe-Ni_2_AlTi micropillars with 1.4 at.% and 3.3 at.% added Mo and with the precipitates outlined. The red lines outline the areas for which *p*_*plastic*_ has been measured. Unit cells and stereograms show the crystallographic orientation and active slip systems in each pillar. The compression axis is shown in green, the slip planes in red and the slip directions in blue. (**c**) The decrease of *p*_*plastic*_ with Mo content (**d**) Stress-strain curves of the compression tests to a nominal compressive strain of 0.1 showing that the high strength of the precipitate-matrix material is maintained.

The mappings of *γ*_*max*_ in compressed micropillars of +1.4 at.% and +3.3 at.% Mo are shown in Fig. 5a and b, respectively. In both cases, the spacing between slip bands is seen to decrease to the value measured earlier in the ferritic matrix micropillar. Also, there are no regions where high strains accumulate in the matrix as observed in the Mo-free microstructure. In fact, *p*_*plastic*_ is found to decrease to 0.0020 in the +1.4 at.% Mo and −0.0006 in the +3.3 at.% Mo, see plot in Fig. 5c. The decrease in plastic strain partitioning did not affect the strengthening provided by the precipitate phase, as shown in the stress-strain curves of the compression tests in Fig. 5d.

**Figure 6:**
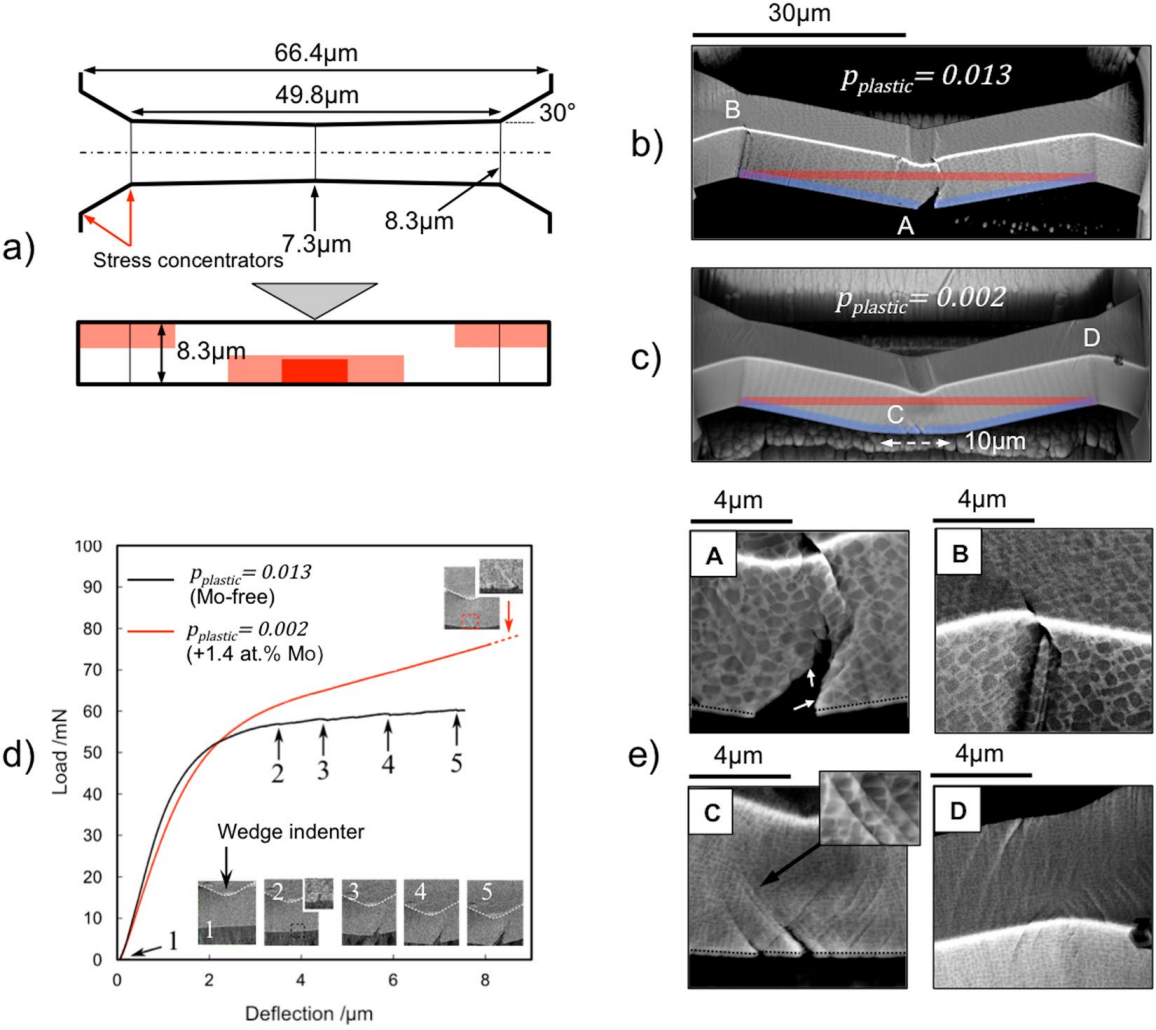
Microbeams bending tests. (**a**) Top and side view of the bowtie geometry. Red areas indicate the regions of maximum tensile stresses. (**b** and **c**) Post-mortem backscatter images acquired at a 52° tilt of the Mo-free and +1.4 at.% Mo microbeams, respectively. Thick lines highlight the differences in length of the bottom region of the microbeams before (red) and after (blue) deformation. (**d**) Compliance-corrected load-deflection curves with inset taken from video frames showing the initiation and propagation of the cracks. (**e**) Details of the backscatter images. (Top) Mo-free. (Bottom) +1.4 at.% Mo showing slip banding. The dashed lines at the bottom edges underline the pulverised material deposited here during the final milling step.

Assuming that Mo addition has a negligible effect on *G*_*M*_, substituting the values of *p*_*plastic*_ and *f* (Table 1) into Equation 6 gives *τ*_*B*_ = 108 MPa for the +1.4 at.% Mo and *τ*_*B*_ = 31 MPa for the +3.3 at.% Mo, indicating that the rate at which the back stresses build up has been lowered. As discussed above, this would prevent early cavitation and cracking in the precipitate-matrix material under tensile loads. To demonstrate this, it is necessary to compare the response of the Mo-free and Mo added alloys to tensile stresses. Here, we use an indirect microtensile testing method^53,54^ in which microbeams are deflected at the centre while clamped at the extremes. The beams therefore elongates during deflection causing an axial tensile stress state to superimpose onto the one associated with pure bending. The three most stressed regions are located at the clamped ends and at the centre, as shown in Fig. 6a. If the cross-section of the beams is constant, however, the milled edges at the ends would act as stress concentrators, causing early damage. The bowtie geometry illustrated in Fig. 6a is therefore adopted to reduce the tensile stresses at the extremes and ensure these build up at the central-bottom region. The technique can then be used to compare the ductility of different materials by estimating the difference in the plastic strain that accumulates in such region before this cracks.

In the case of the +3.3% Mo, the relatively small spacing between the dendrites of the additional Mo-rich phase prevented the testing of microbeams free from hard masses. Being large non-deformable and brittle particles, these led to premature failure of the microbeams. For this reason, only the results of the Mo-free and the +1.4 at.% Mo tests are described hereafter. Results are shown in Fig. 6b–e and videos captured *in-situ* supplied as Supplementary Video S1 and Supplementary Video S2.

In the Mo-free microbeam, cracking occurs in the central region of maximum tensile stresses, as expected, Fig. 6b. A single crack initiates at ~3.5 μm deflection causing a small load drop in the load-deflection curve, point 2 in Fig. 6d, and grows gradually through discrete steps that produce further load drops, points 3, 4 and 5 in the same curve. The crack path is sometimes seen to circumvent the precipitates as in those highlighted by arrows in Fig. 6e (Detail A). A second crack later appears in the tensile zone at the left end of the microbeam (Detail B). In contrast, in the tensile regions of the +1.4 at.% Mo microbeam, sharp slip bands develop which are seen to cut through the precipitates, as shown in Fig. 6e (Details C and D). Cracking initiates only at ~8.4 μm deflection and at 30% higher loads, Fig. 6d. In particular, two cracks are seen to grow along parallel slip bands and stop at the intersection with other (transversal) slip bands, (Detail C), suggesting that the plastic strain that accumulates in the latter has prevented stress localisation ahead of the crack tips.

As volume is conserved during plastic deformation, the accumulation of plastic strain in the central region of maximum axial tensile stresses must be accompanied by a reduction in the cross-section of the beam. This local reduction is not appreciable in the post-mortem SEM images of the Mo-free microbeam, indicating that the residual (axial) plastic strain of the region as a whole is below 0.01. In fact, the length of the bottom part of the microbeam highlighted in Fig. 6b (red line) coincides with its initial length (blue line). In the +1.4 at.% Mo microbeam, the transversal shrinkage is instead significant enough to cause a noticeable flattening of the bottom surface of the beam over a distance of approximately 10 μm, as indicated in Fig. 6c. As the microbeam permanent elongation of 0.8 ± 0.1 μm occurs along this length, the axial strain, *ε*_*axial*_, in the region is of the order of:

7 $${\varepsilon }_{axial}=\frac{{\rm{l}}-{{\rm{l}}}_{0}}{{{\rm{l}}}_{0}}=\frac{10-9.2}{9.2}\simeq 0.09$$

This demonstrates that the Fe-Ni_2_AlTi precipitate-matrix microstructure has acquired the capability of deforming plastically under tensile loads.

## Summary and Outlook

We have shown that strain mapping using nanoscale digital image correlation enables the plastic strain difference between the matrix and the precipitate phases in precipitate-strengthened alloys to be measured. We have then extended the Ashby model to shearable particle so that the measurement of this strain difference, *p*_*plastic*_, could be used to estimate the back stresses at the surface of the precipitates. Their build-up promotes cavitation and leads to premature failure under tensile loads. Consistent with this, we found these stresses to accumulate more rapidly in the Fe-Ni_2_AlTi (precipitate-matrix) microstructure than in the one of the more ductile Ni-based superalloy CMSX-4. With the help of crystal plasticity calculations, we then aimed at finding ways to improve the mechanical behaviour of the former by controlling the value of *p*_*plastic*_. This was achieved without loss in precipitate strengthening by decreasing the lattice misfit between the matrix and the precipitates and, at the same time, increasing the number density of precipitates. We then provided evidence of the enhanced ability of the new Fe-Ni_2_AlTi microstructure to deform plastically under tensile stresses.

A significant increase in failure strains (to ~8% elongation) has been recently observed in precipitate-strengthened maraging steels following the minimisation of lattice misfit and the reduction of the precipitate size and spacing to few nanometres^6^. As the strength was retained, a unique combination of high strength and good ductility was obtained. The authors of the latter study considered the link between this specific microstructure and the exceptional mechanical proprieties to be counterintuitive. Here, we have demonstrated otherwise.

Moreover, our study indicates that reducing lattice misfit and the precipitate size and spacing might not be the only way to create a very strong and yet ductile precipitate-matrix microstructure. The mechanical response of each phase is in fact controlled by a multitude of others physical quantities, such as the antiphase boundary energy in the precipitate phase or the lattice resistance in the matrix phase^38^. These are often difficult to measure and control without affecting other microstructural parameters. Measuring a sufficiently low *p*_*plastic*_ at low temperatures therefore emerges as the way in which one can verify that the above variables have been appropriately balanced to enhance the ductility of the precipitate-matrix microstructure.

## Methods

### Sample preparation and characterisation

The CMSX-4 samples, supplied by Rolls-Royce plc, were heat treated for eight hours at 1314 °C, followed by ageing for four hours at 1140 °C and then 16 hours 870 °C. The Ni_2_AlTi-strengthened ferritic alloy, with a nominal composition Fe-20Ni-13.5Al-13.5Ti (at.%), was prepared by vacuum arc-melting. The bar of material was turned and re-melted a further five times to ensure the composition was homogeneous. The sample was then solution treated under vacuum at 1200 °C for two hours before being water quenched to room temperature. Vacuum arc-melting was also used to prepare the ferritic matrix-only sample with nominal composition Fe-6.5Ni-3.8Al-4.5Ti (at.%) and the alloys with added Mo. The compositions of these materials were verified by energy dispersive X-ray spectroscopy (EDX).

The average size, spacing and volume fraction of the precipitates were estimated from backscatter electron (BE) images of the microstructures using the mean lineal intercept of the precipitates and the mean free path between precipitates. Five large areas micrographs of each sample were considered. The results were in line with those obtained for the pillars, supporting the assumption that the microstructural parameters did not vary significantly into their depth.

Because of the cubic shape of the Ni_3_Al precipitates in the CMSX-4, the BE images of the sides of the samples parallel to the {001} planes were used. The misfit between the matrix and the coherent precipitates for the Fe-Ni_2_AlTi alloys was measured by X-ray diffraction (XRD). Each peak was individually fitted and then used for a least squares linear regression to determine the lattice parameter of each phase. Details can be found in Jones^55^.

### Micromechanical testing

#### EBSD measurements

**Figure 7:**
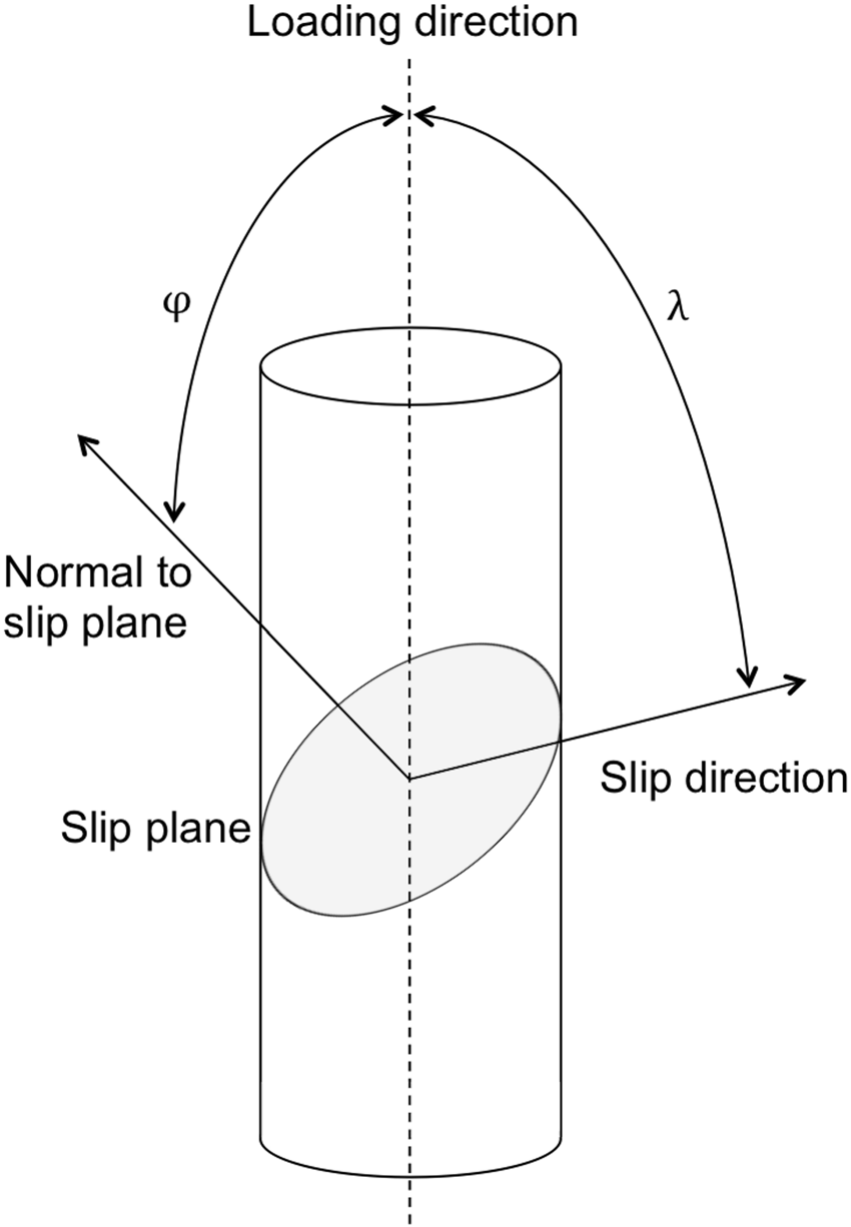
Angles considered in the calculation of the Schmid factor.

Electron backscatter diffraction (EBSD) mapping was used to sample the crystallographic orientation at the edge of the samples where micropillars were to be milled. The locations chosen were those expected to give the least amount of out-of-plane slip, having considered the orientation of the slip system most likely to activate during microcompression. After testing, the crystallographic orientation was used to measure the angle between the slip planes and the plane of investigation in the calculations of the slip band spacing and to derive the critical resolved shear stress, as:

8 $${\tau }_{{\rm{c}}}={m}_{{\rm{\max }}}\times {\sigma }_{{\rm{y}}}.$$

where *σ*_y_ is the yield stress and *m*_max_ the maximum value of the Schmid factor *m* = cos *φ* cos *λ*. As illustrated in Fig. 7, *φ* is the angle between the loading direction and the normal to the slip plane and *λ* is the angle between the loading direction and the slip direction. The {111}〈011〉 slip systems for CMSX-4 and the {011}〈111〉 slip systems for the ferritic steel were considered in the calculation of *m*_max_. The values of the above quantities are given in Table 2.

**Table 2:** Values used in the calculation of the critical resolved shear stresses.

Alloy	Loading direction	*φ* (°)	*λ* (°)	*m*_*max*_	*σ*_y_ (MPa)	*τ*_c_ (MPa)
CMSX-4 [010]	[0 1 0]	54.7	45	0.408	1000	**410**
CMSX-4 [011]	[8 61 79]	40.4	52.1	0.468	1100	**510**
Fe-Ni_2_AlTi	[56 4 83]	52.1	38.9	0.478	1500	**720**
+1.4 at.% Mo	[2 1 100]	44.4	54.3	0.416	1600	**670**
+3.3 at.% Mo	[13 69 71]	53.5	42.8	0.437	2300	**1000**
Ferritic matrix	[25 15 96]	51.8	38.5	0.484	1000	**480**

#### Micropillar preparation

Square section pillars with a side length of 5 μm and an aspect ratio of approximately 1:3 were milled at the edge of each sample, using a focussed ion beam scanning electron microscope (FIB SEM) (Helios Nanolab, FEI, USA). The pillar was positioned ~5 μm from the edge of the sample to avoid any overhang. Initially, the material was milled rapidly, at a current of 9 nA and an accelerating voltage of 30 kV, to shape the pillar and remove surrounding material. The final step involved a cleaning cross-section at 0.43 nA and a 2° offset from the vertical to remove any taper. The face on which the pattern was to be deposited was milled last, at a current of 0.23 nA, to reduce Ga+ implantation and re-deposition from the FIB milling. Finally, the sample was orientated so that the top of the pillar could be milled, to ensure it was flat and normal to the pillar axis. The above milling sequence helps minimise the effect that tapering and ion damage have on the observed strain hardening, an effect which is nevertheless expected to be negligible for the relatively large pillars tested here^56,57^.

#### Speckle patterning

With the sample orientated so that the face for Pt deposition was normal to the electron beam, the speckle pattern was reproduced from the bitmap image provided in Di Gioacchino and Clegg^17^. The pattern, containing 4000 speckles of 3 × 3 pixels, was deposited at the top of the pillar over an area of 4 × 8 μm^2^. The deposition was completed in a single pass to avoid the spreading of deposited material that follows possible shifts in beam position due to sample charging. The amount of material deposited per point was controlled by the dwell time and was chosen to produce speckles ~40 nm in diameter. The conditions used are given in Table 3.

**Table 3:** Electron beam deposition settings.

Voltage/kV	Current/nA	Dwell time/μs	Passes/No.	Duration/s	Beam size/nm
15	1.4	1200	1	43	13.3

#### Micropillar testing

An image was acquired before compression, using the settings in Table 4. Microcompression was then carried out with an *in situ* nanoindenter using a 10 μm flat punch (Alemnis, CH) in a field emission gun scanning electron microscope (FEG SEM) (Cross beam, Zeiss, GE). The punch was brought into proximity with the pillar and the latter compressed to ~0.1 strain with a nominal rate of 5 × 10^−3^ s^−1^. An image of the deformed pillar was taken in the original FIB-SEM microscope under the same conditions for DIC.

**Table 4:** SEM imaging settings.

Voltage/kV	Current/nA	WD/mm	Scan time/min	Image size/pixels	Res/nm
15	1.4	4	6.1	2048 × 1768	~5

#### Microbeam preparation and testing

The same FIB SEM used for fabricating the micropillars was used to mill the bowtie-shaped microbeams. After an initial cut at 21 nA, four milling steps at 2 nA were alternated between the top and side to minimise the (re)deposition of the pulverized material on the edges of the beams. The microbeams were deflected at the centre at a nominal rate of 0.1 μm s^−1^ using a 10 μm long wedge-shaped tip oriented transversally to the beam.

#### Strain mapping

Digital image correlation was carried out using commercial software (DaVis, LaVision, Germany), using subsets (S) down to 16 × 16 pixels giving a spatial resolution of up to 80 × 80 nm^2^, with a window overlap of 50%. The image of the deformed pillar was correlated with the undeformed pillar image to obtain the 2D displacement field on the *xy* plane of investigation. This was used to calculate the maximum shear strain, γ_*max*_, at each subset:

9 $${{\rm{\gamma }}}_{max}({\rm{S}})=2\cdot \sqrt{{(\frac{{\varepsilon }_{xx}-{\varepsilon }_{yy}}{2})}^{2}+{(\frac{{\gamma }_{xy}}{2})}^{2}}$$

where *ε*_*xx*_, *ε*_*yy*_ and *γ*_*xy*_ = 2*ε*_*xy*_ = *ε*_*yx*_ are the strain components in the *xy* plane. Note that γ_*max*_ is sometimes defined as the square root term alone.

In the case of simple shear depicted in Fig. 3 to illustrate the Ashby model, in which the slip direction is in the *xy* plane, *ε*_*xx*_ − *ε*_*yy*_ =0 and hence *γ*_*max*_ ≡ *γ*_*xy*_. As described above, the crystallographic orientations of the micropillars were chosen as to minimise out-of-plane slip. Nevertheless, where the latter occurs, it would manifest in the DIC measurements as an apparent in-plane stretch (or compression), i.e. generally *ε*_*xx*_ − *ε*_*yy*_ ≠ 0. Therefore, the expression for γ_*max*_ in Equation 9 has been used here and in previous high-resolution DIC studies^26,33,58^ because it compensates for the lack of out-of-plane displacement data.

Two images of the same speckle pattern acquired at the conditions used for testing were correlated to characterise the spatial distortion associated with irregularities in the SEM scan. The correlation produced a bundle of horizontal bands of ~0.04 strain in the γ_*max*_ map. Therefore, each individual value had an error of *δ* = ±0.04.

#### Splitting of the strain data

Images of the pillars used for DIC also displayed compositional contrast, as shown in Fig. 1. This enabled the positions of the precipitates to be identified and outlined directly on the maps of maximum shear strain. The outlining was done by inspection by eye and thresholding on the ImageJ software (National Institute of Health, USA). The value of $\langle {\gamma }_{max}^{P}\rangle$ was determined by summing the maximum shear strain values obtained for the precipitate phase (P) using Equation 9 and dividing by the number of data points, *N*^*P*^:

10 $$\langle {\gamma }_{max}^{P}\rangle =\frac{{\sum }_{S{\epsilon }P}{\gamma }_{{\max }}(S)}{{N}^{P}}$$

The same was done to calculate $\langle {\gamma }_{max}^{M}\rangle$.

The standard error in the mean shear strain was then calculated as ${\rm{S}}.{\rm{E}}.=\delta /\sqrt{n}$, where *n* is the number of shear strain values used to calculate the mean. For each phase, the mean shear strain was calculated from a large number shear strain values (>2000) and therefore the corresponding error was relatively small <0.0008. Despite this uncertainty, it is important to note that the sign of the calculated *p*_*plastic*_ remains unaffected.

### CPFEM: meshing, boundary conditions and calibration

**Figure 8:**
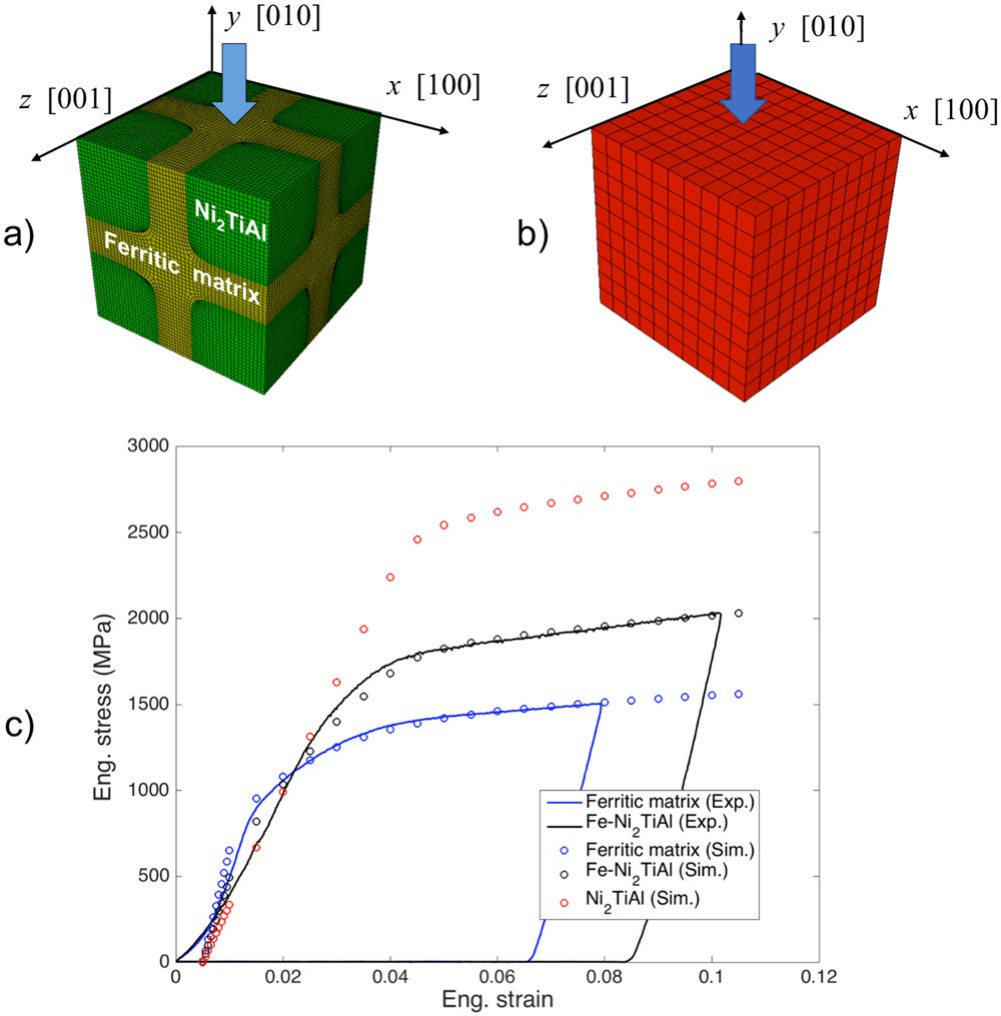
(**a**) Two-phases FE model used to represent a unit volume of the Fe-Ni_2_AlTi microstructure. (**b**) Single-phase FE model. (**c**) Fitting of the experimental stress-strain curves (ferritic matrix-only and Fe-Ni_2_AlTi micropillars) and predicted mechanical response of the Ni_2_AlTi precipitate phase.

The finite elements domain considered in the present study is a cube having one-eighth of a precipitate in each corner and the faces parallel to the {001} directions, Fig. 8a. The cuboidal precipitate was reproduced by 163,400 hexahedral finite elements, and the smooth interfaces between two phases were constructed while maintaining precipitate volume fraction of 50.6%^59^. Taking an orthogonal (*x*, *y*, *z*) coordinate system with origin at a top corner of the cube and axes normal to its faces, as shown in the same figure, the compression was applied with a rate of 1 × 10^−3^ s^−1^ by displacing the *y* = 0 face along -*y* direction while imposing *u*_*x*_ = 0 on the *x* = 0 face, *u*_*z*_ = 0 on the *z* = 0 face and *u*_*y*_ = 0 on the -*y* face. The {011}〈111〉 slip systems were considered. No initial internal stresses were imposed at the precipitate-matrix interface.

The slip rate on each slip system was represented as a power-law function of the resolved shear stress. A dislocation density based hardening was also adopted^60,61^. The hardening parameter, *g*, has the expression:

11 $$g=AGb\sqrt{\sum _{\alpha =1}^{N}\,{\rho }^{\alpha }}$$

where *A* is a material constant, *G* is the shear modulus, *b* is the Burger’s vector and *ρ*^*α*^ is the dislocation density in α-th slip system. The evolution of dislocation density is then obtained by a standard phenomenological equation,

12 $${\dot{\rho }}^{\alpha }=({k}_{1}\sum _{\beta =1}^{N}\,{\rho }^{\beta }-{k}_{2}{\rho }^{\alpha })\cdot |{\dot{\gamma }}^{\alpha }|$$

where, *k*_1_ and *k*_2_ control the generation and annihilation of dislocations, respectively.

In the present study, values of *k*_1_ and *k*_2_ for the matrix phase were acquired by fitting the stress-strain curve predicted for a “single phase” cuboid, Fig. 8b, to the curve obtained for the ferritic matrix micropillar. The hardening parameters for the Ni_2_AlTi phase were then adjusted to make the stress-strain curve predicted for the “two phases” FE cuboid match that of the Fe-Ni_2_AlTi micropillar. The two fitted stress-strain curves and the one predicted for the Ni_2_AlTi material are shown in Fig. 8c.

The values of the parameters used for the CP-FE calculations are reported in Table 5.

**Table 5:** Values of the parameters used in the CP-FE analysis.

	k_1_	k_2_	C_11_/GPa	C_12_/GPa	C_44_/GPa	b/mm
Ni_2_AlTi	1.5 × 10^8^	3 × 10^2^	152.0	103.9	94.0	2.29 × 10^−7^
Fe Matrix	1 × 10^8^	3.5 × 10^2^	228.0	132.0	116.5	2.48 × 10^−7^
Matrix 1	1.4 × 10^8^	3.5 × 10^2^	”	”	”	”
Matrix 2	5 × 10^8^	3.5 × 10^2^	”	”	”	”
Matrix 3	2 × 10^9^	1 × 10^3^	”	”	”	”

## Electronic supplementary material

Supplementary Video S1

Supplementary Video S2
